# bZIPs and WRKYs: two large transcription factor families executing two different functional strategies

**DOI:** 10.3389/fpls.2014.00169

**Published:** 2014-04-30

**Authors:** Carles M. Llorca, Maren Potschin, Ulrike Zentgraf

**Affiliations:** Department of General Genetics, Center of Plant Molecular Biology, University of TübingenTübingen, Germany

**Keywords:** bZIPs, WRKYs, DNA-binding, heterodimerization, regulatory mechanisms, G/C box accumulation, W-box accumulation

## Abstract

bZIPs and WRKYs are two important plant transcription factor (TF) families regulating diverse developmental and stress-related processes. Since a partial overlap in these biological processes is obvious, it can be speculated that they fulfill non-redundant functions in a complex regulatory network. Here, we focus on the regulatory mechanisms that are so far described for bZIPs and WRKYs. bZIP factors need to heterodimerize for DNA-binding and regulation of transcription, and based on a bioinformatics approach, bZIPs can build up more than the double of protein interactions than WRKYs. In contrast, an enrichment of the WRKY DNA-binding motifs can be found in WRKY promoters, a phenomenon which is not observed for the bZIP family. Thus, the two TF families follow two different functional strategies in which WRKYs regulate each other’s transcription in a transcriptional network whereas bZIP action relies on intensive heterodimerization.

## INTRODUCTION

Due to their sessile nature, plants cannot move to avoid unfavorable conditions as animals do, thus they are forced to cope with their immediate environment, whatever this is. Since the potential environmental variability covers a continuum range from the optimal growth conditions to the toughest stress, a complementary number of possible physiological responses have evolved in order to respond in the most convenient manner to any possible scenario. This process involves transcription factor (TF) networks modulating the expression of a huge number of responding genes.

Unraveling how these networks operate is a major field in plant research, since the comprehensive understanding of the regulatory circuits will allow us to modify them in a beneficial way in the current context of growing food demand and global climate change. Many efforts are focused on deciphering the structure of specific networks by identifying up- and downstream components, however, the comparative analysis of the general features of the regulation of whole families of TFs is still challenging. Granted that TFs within the same family are evolutionary closely related, they are likely regulated by common mechanisms. The recognition of these strategies, shared by entire families of TFs, can provide useful clues to better characterize the function of members of these families.

In this review, we summarize the major regulatory mechanisms characterized so far for WRKYs and bZIPs, two of the largest TF families in plants. Although they have a comparable size, 75 bZIPs and 76 WRKYs can be found in the TAIR database, and they regulate critical physiological processes, such as plant defense, stress responses, or development including senescence; they appear to follow different regulatory strategies. Whereas WRKYs are strongly regulated at the transcriptional level by each other, bZIPs are regulated predominantly at the post-translational level via the formation of heterodimers. This distinction can be inferred from a bioinformatics approach whereby all the *Arabidopsis* bZIPs and WRKYs IDs gathered from the TAIR were used as an input for the *Arabidopsis* Interaction Viewer in the BAR webpage (http://bar.utoronto.ca/welcome.htm). The 76 WRKYs resulted in 170 interactions, while the 75 bZIPs yielded in 389, more than the double than WRKYs. In addition, the WRKY binding motifs (W-boxes) are found to be enriched in the WRKY gene promoters compared to the average occurrence over all *Arabidopsis* genes. In comparison, C- and G-boxes, the preferred bZIP binding motifs in plants, are not enriched in the bZIP promoters.

## THE bZIP TFs AND THEIR REGULATION

This family of dimeric TFs is present in all eukaryotes, from *Saccharomyces cerevisiae* (17 bZIP genes) to human (56 bZIP genes). bZIPs have been described in *Arabidopsis* (75), rice (89), sorghum (92), soybean (131), and recently in maize (125; Wei et al., 2012). In plants, they are involved in important processes such as pathogen defense (Alves et al., 2013), abiotic stress signaling (Fujita et al., 2005), hormone signaling (Choi et al., 2000), energy metabolism (Baena-González et al., 2007), as well as development, including flowering (Abe et al., 2005), senescence (Smykowski et al., 2010), and seedling maturation (Alonso et al., 2009).

The name of the family is derived from the basic region/leucine zipper (bZIP) domain present in all its members. This domain consists of an uninterrupted α-helix comprising a basic region (BR) which is necessary and sufficient to bind the DNA, followed by a C-terminal leucine zipper (LZ) motif responsible for the dimerization (Schumacher et al., 2000; Miller et al., 2003). The bZIP family was subdivided according to sequence similarities and functional features resulting in 10 groups named A to I, plus S in *Arabidopsis* (Jakoby et al., 2002; Nijhawan et al., 2007; Wei et al., 2012). While many bZIPs can form homodimers, bZIP members classified in different groups can be combined through heterodimerization to form specific bZIP pairs with distinct functionalities.

### THE bZIP STRUCTURE DETERMINES THE DIMERIZATION SPECIFICITY

By means of dimerization, a limited number of monomers can generate a wide pool of different dimers with singular properties, thereby expanding the repertoire of regulatory responses (Amoutzias et al., 2006, 2008). However, protein interaction has to be selective in order to grant the appropriate response to each situation. In agreement to that, Newman and Keating (2003) showed that, in human and yeast, only 15% of all possible interactions actually take place between bZIP proteins. This specificity relies on the constitution of the LZ, which is composed of structural repetitions of the so called heptads. In each heptad, seven amino acids are arranged around two α-helix turns, in which two definite positions are occupied by leucines or other hydrophobic amino acids. These residues expose their side chains to the same side of the helix, thus resulting in an amphipathic structure. Based on this conformation, hydrophobic forces created between the non-polar sides of two LZs drive their dimerization (Vinson et al., 2002). However, the remaining composition of the heptad is decisive in determining if the interaction will actually take place.

**FIGURE 1:**
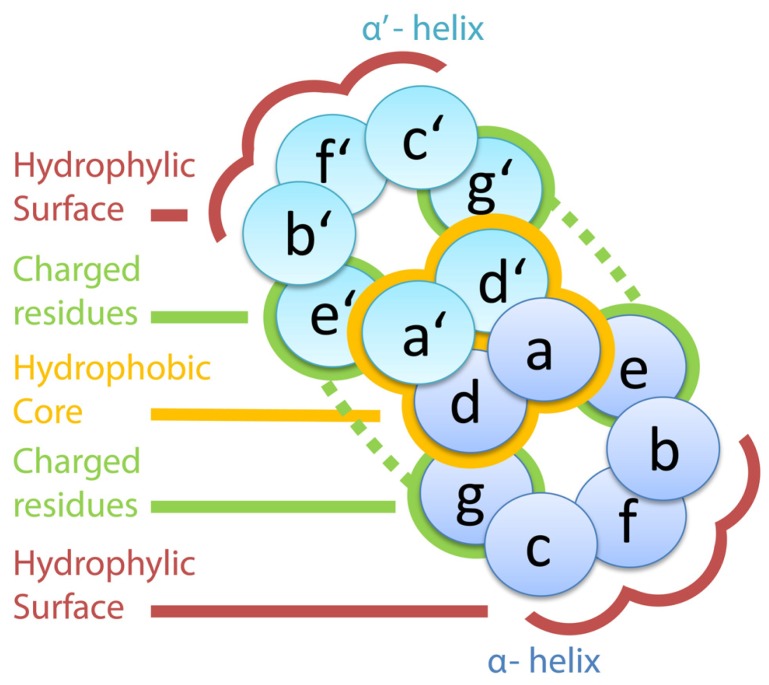
**Outline of a section through two a helices interacting via leucine zippers.** The amino acids in positions ***a*** and ***d*** configure the hydrophobic core, which is indicated with the yellow halo. Charged residues in positions ***e*** and ***g*** generate electrostatic forces, represented by the dashed green lines. The hydrophilic surface is formed by the amino acids in positions ***b***, ***c***, and ***f***.

Understanding the forces governing the bZIP dimerization has been a field of intensive research in recent years. To this end, the amino acid positions within a heptad are designated by a specific nomenclature with a letter ranging from ***a*** to ***g*** (Deppmann et al., 2006). According to that, positions ***d*** and ***a*** carry the hydrophobic residues and define the hydrophobic face; whereas positions ***b***, ***c***, and ***f*** are located on the opposite side, the hydrophilic one (**Figure 1**). Based on this codification and the already described interactions, rules governing the interaction have been formulated (Vinson et al., 2002) and even methods for dimer predictions have been created (Fong et al., 2004). Accordingly, the amino acids in positions ***a***, ***d***, ***e***, and ***g*** are the ones with a greater impact on determination of the specificity of the interaction (Deppmann et al., 2004). First, the primary hydrophobic forces are established between ***a*** and ***d*** positions of a heptad and their counterparts in the other LZ disposed in parallel (Vinson et al., 2002), in which the presence of leucines in the ***d*** positions is the most stabilizing factor for the dimerization (Moitra et al., 1997). Next, ***a***–***a′*** interactions contribute in determining the homodimerizing partners: asparagine residues in this position tend to interact rather with another asparagine, thus favoring the homodimer formation. Conversely, if this position is occupied by a lysine or serine, the heterodimer is favored as these two amino acids prefer residues other than themselves (Acharya et al., 2002). In addition, positions ***e*** and ***g*** are stabilizing the helix. These two positions act crosswise, so that ***e*** positions of one helix interact with ***g*** positions on the other one, and usually carry charged or polar amino acids. As a consequence, depending on the charge of these residues, attractive or repulsive forces are formed between the two LZs (Krylov et al., 1994). Overall, the amino acid composition of the LZ determines the energy of the interaction, making each dimer combination more or less likely to happen (Vinson et al., 2006).

Under the above mentioned rules, *Arabidopsis* bZIPs are predicted to form, almost exclusively, homodimers or quasihomodimers (dimers between two paralogs; Deppmann et al., 2006). Dimerization between bZIPs belonging to the G group (Shen et al., 2008), H group (Holm et al., 2002) or A group (Bensmihen et al., 2002) are in agreement with these predictions. In addition, bZIP are also able to heterodimerize specifically, as the following examples illustrate. The E group members bZIP34 and bZIP61 are unable to homodimerize due to the presence of a proline residue in their LZ, nevertheless they do form heterodimers with the bZIP51 (I group) or the bZIP43 (S group; Shen et al., 2007); G-box binding factor 4 (GBF4), belonging to the A group, interacts with members of the G group (Menkens and Cashmore, 1994); members of the H group can heterodimerize with the G group bZIP GBF1 (Babu Rajendra Prasad et al., 2012 ); or even a whole heterodimerization network involving bZIPs from C and S groups has been described (Ehlert et al., 2006).

The preference of bZIPs to interact with more related partners reflects the selectivity of the dimerization. Because they perform similar, even overlapping, functions and can bind to the same *cis*-elements (Jakoby et al., 2002); they can interact laxly, for their ultimate function is not altered to a great extent. In contrast, heterodimerization between bZIPs which are more evolutionary distant is more restricted, as it brings together monomers with more disparate properties. Therefore, the specificity of the partner selection is of central importance because the composition of the dimer will define decisive functionalities such as transactivation potential or DNA-binding activity.

### THE bZIP DIMER COMPOSITION DETERMINES THE DNA BINDING

**FIGURE 2:**
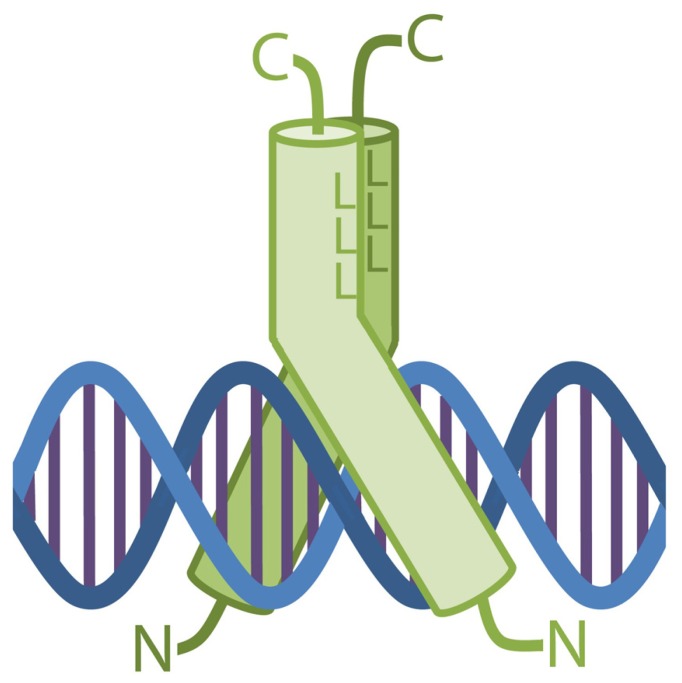
**Schematic drawing of a bZIP dimer bound to the DNA.** The two proteins form a Y-shape structure which embraces a perpendicularly disposed DNA molecule. The major groove is contacted by both bZIPs via their DNA-binding domains. L represents the leucines forming the interface in the bZIP dimer.

The DNA recognition by bZIPs takes place with the two continuous α-helices wrapped around their LZ regions and pulled apart slightly on their N-terminus, forming a Y-shaped structure which embraces the DNA duplex (**Figure 2**). In this complex, each BR contacts the DNA along the major groove on opposite sides of the double helix, so that each monomer binds one-half of the DNA target sequence (Glover and Harrison, 1995). As a consequence of the dimeric arrangement of the bZIPs, the binding properties of each dimer are determined by its singular monomer composition.

The target sequences preferentially bound by bZIPs are palindromic or pseudo-palindromic hexamers with an ACGT core (Foster et al., 1994). The positions within a hexamer are designated with a number as established by Oliphant et al. (1989). Under this code, the bases are given a number radiating from the central positions, i.e., CG, which are both referred as 0. So, the 5′ half of the sequence are negative values, while the 3′ half are positive. Based on the nucleotide position +2, different kinds of ACGT-containing elements are classified as A-box, C-box, G-box, or T-box; among which C and/or G-boxes are preferentially bound by plant bZIPs (Izawa et al., 1993). Furthermore, the protein binding affinity is determined to a great extent by the nucleotides flanking the hexamer (Williams et al., 1992). The specificity of the DNA recognition arises, thus, as a result of variations in the *cis*-element sequence combined with the existence of unique BRs mixtures able to discriminate them.

Where exactly the specificity of the interaction relies on has been revealed from solved structures of bZIPs bound to DNA. The target sequence is contacted by only five residues in each BR all along 12 bp in the major groove and these contacts are extended by water molecules (Fujii et al., 2000). These key positions form part of an invariant sequence of nine amino acids (N-X7-R/K) which feature the BR. Granted that the BR is the most conserved region in bZIPs, the binding preferences of each monomer are determined by only subtle differences in its sequence. For instance, in mouse, bZIPs belonging to the CCAAT/enhancer-binding proteins (C/EBP) family, carry a valine residue in the position 5 of the signature sequence which discriminates against purines at position -3 of the DNA binding site (Miller et al., 2003). Therefore, unspecific interactions with similar DNA sequences are prevented by this single residue. In another case, in the AP-1-like TF (YAP) and CAMP response element binding protein-2 (CREB2) subfamilies of bZIPs in *Schizosaccharomyces pombe*, the presence of a hydrophobic residue in position 8 of the invariant sequence favors the contact with their AT-rich binding site targets (Fujii et al., 2000).

Besides the identity of amino acids directly contacting the DNA, the specificity of the DNA recognition is further modified by functional variability of the amino acids in the BR. This means that they can adopt different conformations depending on the accompanying residues, which creates a different set of contacts with the DNA bases (Miller et al., 2003). Beyond the BR, it is also known that the hinge region, the junction between the LZ and the BR, participates in determining the DNA-binding specificity (Niu et al., 1999). Likewise, the presence of ions between the dimer and the DNA (Schumacher et al., 2000), the redox status (Shaikhali et al., 2012), or even the DNA flexibility (Konig and Richmond, 1993) also affect the DNA-binding. On top of that, the BR is intrinsically unstructured in absence of DNA and the folding is only induced upon association with the double helix (Seldeen et al., 2008). Such lack of definite conformation allows the interaction with multiple *cis*-elements and facilitates post-translational modifications by better exposing the lateral chains, enhancing the regulatory possibilities (Dyson and Wright, 2005).

Granted that the specificity of the DNA recognition arises from the contribution of each BR individually, heterodimerization determines the manner in which the bZIP pairs recognize their target sequences. Through specific heterodimer formation, for example, the binding activity of bZIP53 to the albumin 2S2 promoter is significantly enhanced when combined with bZIP25 or bZIP10 (Alonso et al., 2009). Conversely, other bZIPs lose their DNA-binding when associated to particular partners, as bZIP1, whose DNA-binding activity is prevented in combination with bZIP63 or bZIP10 (Kang et al., 2010).

### THE DIMER COMPOSITION DETERMINES THE TRANSACTIVATION PROPERTIES

In addition to their role in the DNA recognition, each monomer contributes individually to the transactivation capacity (Miotto and Struhl, 2006). While some bZIPs have special domains acting as transactivators or as repressors, e.g., the proline rich domain in the G group (Shen et al., 2008), others require the presence of additional elements, such as coactivators (Rochon et al., 2006) or histone deacetylases (Kuo et al., 2000). Besides, the transactivation activity of the same bZIP can be further modified through the interaction with other proteins, e.g., the transactivation capacity of ELONGATED HYPOCOTYL 5 (HY5) is enhanced by the clock protein CCA1 (Andronis et al., 2008), but is inhibited when interacting with BBX25 (Gangappa et al., 2013). As a result of the peculiar transactivation properties of each bZIP, the composition of the dimer determines the outcome of target gene expression.

More important, if different dimers can affect the expression in a peculiar manner, they can compete for the same *cis*-element with other bZIP pairs, constituting an efficient mechanism to adjust the expression of a given gene. Such a system has been described controlling the expression of late-embryogenesis abundant genes in *Arabidopsis* by the A group bZIPs ABA-insensitive 5 (ABI5) and EEL. These two bZIPs compete for the same binding site, conferring antagonistic transactivation functions: ABI5 homodimers activate the gene expression, whereas EEL homodimer and ABI5–EEL heterodimer repress it (Bensmihen et al., 2002). Furthermore, gradation of the expression can be achieved through the formation of different heterodimers. So is the expression of RBCS1a modulated by HY5, HY5 homolog (HYH), and GBF1 in which GBF1 acts a repressor, HY5 and HYH act as inducers. However, the different heterodimers that can be formed show intermediate effects depending on the pair of monomers combined (Singh et al., 2012). In other cases, functional cooperation between monomers is established instead of competition. Indeed, heterodimerization appears to be a requirement for the induction of genes under control of bZIPs belonging to the C/S1 network. In other words, while these bZIPs are not able to activate the gene expression by themselves alone, certain heterodimers result in a strong activation of specific target genes (Weltmeier et al., 2006).

### MONOMER AVAILABILITY IS A HOT SPOT IN bZIP REGULATION

Having established how relevant the identity of the monomers in each dimer is, the availability of monomers arises as a key point of regulation restricting the number of interactions that can take place. The expression of bZIP genes is, indeed, adjusted to control their abundance, showing tissue specificity (Fujita et al., 2005; Iwata et al., 2008; Weltmeier et al., 2008; Alonso et al., 2009), as well as developmentally regulated expression, including embryogenesis (Bensmihen et al., 2002; Weltmeier et al., 2008), flowering (Abe et al., 2005), or senescence (Breeze et al., 2011). Furthermore, changes in expression of bZIPs have been reported upon exposure to certain stresses. For example, Zn deficiency increases the transcription of bZIP23 and bZIP19 (Assunção et al., 2010), bZIP53 and bZIP10 are induced after an osmotic stress period (Weltmeier et al., 2006). The ABA-responsive element binding protein (AREB) subfamily of bZIPs is up-regulated by drought and salt in *Arabidopsis* (Uno et al., 2000) as well as in tomato (Hsieh et al., 2010; Orellana et al., 2010). Besides, a remarkable amount of studies relate changes in the bZIP expression to the energy status. These include the repression of bZIP1 and bZIP63 by sugars (Kang et al., 2010; Matiolli et al., 2011) and bZIP11 by darkness (Rook et al., 1998), or the induction of the expression of several bZIPs by the activation of the energy deficiency-related kinase SNF1-related protein kinase 1 (SnRK1; Baena-González et al., 2007). Beyond the transcriptional level, bZIPs are regulated by alternative splicing (Zou et al., 2007) and by controlling the translation initiation, e.g., the repression of translation by sucrose in bZIPs belonging to the subgroup S1 in *Arabidopsis* (bZIP1, bZIP2, bZIP11, bZIP44, bZIP53; Wiese et al., 2004; Weltmeier et al., 2008).

After the protein synthesis, specific control of the protein turnover has been found regulating the abundance of some bZIPs such as GBF1 (Mallappa et al., 2008), ABF1 and ABF3 (Chen et al., 2013), or TGAs (Pontier et al., 2002). In addition, the amount of functionally active monomers in the nucleus is regulated by subcellular partitioning. In order to be targeted to the nucleus, bZIPs carry a nuclear localization signal (NLS) which is located within the BR, overlapping with the invariant DNA binding sequence and consisting of two clusters of lysines/arginines (Miller, 2009). Nevertheless, few bZIPs have been found outside of the nucleus being retained by different means. For instance, bZIP10 is retained in the cytoplasm by the zinc-finger protein lesions simulating disease resistance 1 (LSD1). This protein interferes with the NLS-mediated nuclear import of bZIP10 (Kaminaka et al., 2006). In other cases, bZIPs are actively shuttled out of the nucleus due to the presence of a nuclear export signal (NES; Tsugama et al., 2012) and they stay in the nucleus only when the NES gets masked (Li et al., 2005). Finally, extra-nuclear retention can be achieved by attachment to membranes. The so called membrane associated bZIPs are anchored via an N-terminal trans-membrane domain and are transferred to the nucleus after proteolytic cleavage. In *Arabidopsis*, bZIP17 (Liu et al., 2008), bZIP28 (Liu et al., 2007a), and bZIP60 (Iwata et al., 2008) have been found to be membrane associated so far.

### bZIP ACTIVITY IS BROADLY REGULATED BY PHOSPHORYLATION

The activity of the available bZIP monomers can be further regulated by phosphorylation. This kind of post-translational modification can modify all the above-mentioned mechanisms controlling the TF function. First, dimerization specificity can be altered through phosphorylation of the LZ (Lee et al., 2010). Next, the DNA-binding of the bZIPs to their target sequences can be prevented by the addition of a phosphate group into the BR, which contributes with a negative charge creating repulsive forces with the DNA molecule (Deppmann et al., 2003; Kirchler et al., 2010). Besides, phosphorylation within other regions of the protein can trigger conformational changes required for the activation of the protein (Lee et al., 2010). In addition to the direct effect on the bZIP activity, phosphorylation can adjust the monomer abundance by altering the protein turnover. For example, phosphorylation of ABF3 creates a binding site for a 14-3-3 protein which protects ABF3 from rapid turnover (Sirichandra et al., 2010). Likewise, phosphorylation of HY5 prevents its degradation by impeding the interaction of this bZIP with the E3-ubiquitin-protein ligase COP1 (Hardtke et al., 2000). Finally, phosphorylation can control the bZIP subcellular localization, targeting a bZIP either for nuclear import (Djamei et al., 2007) or for cytoplasmic retention (Ishida et al., 2008).

Above all, the manner in which the activity of a bZIP is regulated is specific meaning that the same kinase enhances the activity of some bZIPs, but diminishes the action of others. Such a situation has been described for instance for EmBP-2 and ZmBZ-1 phosphorylated by CKII (Nieva et al., 2005). The specific effect of the phosphorylation for each bZIP allows the customized regulation of multiple genes by the action of few upstream kinases. This is an optimal feature for the control of responsive pathways and, indeed, bZIPs are frequently found to be involved in such networks like, e.g., the deciphered ABA-responsive pathway in rice, which involves the action of a SnRK, namely SnRK2, activating the transcription of the ABA responsive genes through the phosphorylation of the bZIP proteins OREB1 and TRAB1 (Kagaya et al., 2002; Kobayashi et al., 2005; Chae et al., 2007). Similarly, bZIPs belonging to the S and C groups coordinate the activation of the metabolic response to low energy stress in combination with SnRK1 (Baena-González et al., 2007; Hummel et al., 2009; Dietrich et al., 2011; Cho et al., 2012).

## THE WRKY TFs AND THEIR REGULATION

The WRKY TF family is found in the plant kingdom and belongs also to the 10 largest families of TFs in higher plants. Like bZIPs, the WRKY family is divided into different subgroups, but in contrast to the ten bZIP groups, the WRKY family is only divided into three groups. WRKY factors are also found in the unicellular eukaryote *Giardia lamblia* and the slime mold *Dictyostelium discoideum* (Ulker and Somssich, 2004; Zhang and Wang, 2005), but there is no hint that WRKY TFs exist in animals. However, former analyses have shown that WRKY TFs belong to a WRKY-GCM1 (glial cell missing 1) superfamily which is a widespread eukaryote-specific group of TFs (Babu et al., 2006).

Almost two decades have already passed since their discovery (Ishiguro and Nakamura, 1994; Rushton et al., 1995, 1996) and by now a lot of different functions have been attributed to the WRKY TFs. They participate in the regulation of many plant processes including the responses to pathogen infestation (Pandey and Somssich, 2009; Birkenbihl et al., 2012; Hu et al., 2012; Chujo et al., 2013), abiotic stresses (Jiang and Deyholos, 2009; Rushton et al., 2010; Scarpeci et al., 2013; Wang et al., 2013), trichome development (Johnson et al., 2002), and senescence (Zentgraf et al., 2010; Zhou et al., 2011; Besseau et al., 2012). Northern blot analysis revealed that in *Arabidopsis* around 70% of the *WRKY* genes were differentially expressed in plants after infestation with an avirulent strain of the bacterial pathogen *Pseudomonas syringae* or treatment with salicylic acid (Dong et al., 2003) emphasizing their importance in pathogen response. A more recently described physiological activity of WRKY factors is their participation in the biosynthesis of alkaloids (Suttipanta et al., 2011; Yamada and Sato, 2013; Yang et al., 2013).

### WRKY STRUCTURAL FEATURES

The WRKY factors are named after their characteristic DNA-binding domain (DBD) of approximately 60 amino acids. This domain contains a highly conserved WRKYGQK motif at the N-terminus and a zinc-finger structure at the C-terminus called the WRKY domain. There are two possibilities how the zinc-finger structure of this domain can be formed, either Cx_4-5_Cx_22-23_HxH (C2H2) or Cx_7_Cx_23_HxC (C2HC), in which the cysteine and histidine residues bind one zinc atom and generate a finger like structure. Both, the WRKYGQK motif and the zinc-finger structure are necessary for the DNA-binding activity of WRKY TFs. Mutations in the invariable WRKYGQK motif significantly reduced the DNA-binding activity and substitutions of the conserved C and H residues of the zinc-finger even abolished the DNA-binding (Maeo et al., 2001).

All WRKY proteins contain one or two of these DNA-binding WRKY domains and are categorized into three subgroups dependent on their number of WRKY domains and the zinc-finger structure. Group I WRKY proteins are marked by two WRKY domains with a C2H2 zinc-finger structure. Group II and III WRKY proteins consist of only one WRKY domain with a C2H2 and a C2HC zinc-finger structure, respectively. The group II WRKY proteins were originally further divided into IIa, IIb, IIc, IId, and IIe based on their primary amino acid sequence, but later, phylogenetic analyses have shown, that the subgroups IIa and IIb are combined to IIa + b, and IId and IIe to IId + e (Eulgem et al., 2000; Zhang and Wang, 2005; Rushton et al., 2010).

Recently, it was shown for *Solanum lycopersicum* that even sequence variants for the highly conserved WRKYGQK motif exist. WRKYGKK is the most common variant, but WRKYGMK, WSKYGQK, WQKYGQK, and WIKYGEN have also been described. Furthermore, it was found that also novel zinc-finger variants exist, namely Cx_29_HxH and Cx_7_Cx_24_HxC (Huang et al., 2012). Moreover, Mangelsen et al. (2008) could also detect variants of the WRKYGQK motif (WRKYGKK, WQKYGQK, WRKYGEK, and WSKYGQM) in *Hordeum vulgare*.

The WRKY domain binds to a so called W-box (TTGACC/T) in the promoters of target genes. This sequence is the minimal core element necessary for binding of a WRKY protein to DNA (Rushton et al., 1996; Ciolkowski et al., 2008). W-boxes can be found in the promoters systemic acquired resistance related (SAR) genes, including *isochorismate synthase 1*, *non-expressor of PR genes 1*, and *pathogenesis related 1* (Fu and Dong, 2013); or ABA signaling-related genes such as *ABI4*, *ABI5*, and *ABA responsive element binding factor 4* (Rushton et al., 2012). Often there are several W-boxes in one promoter, and even motif clusters can be found. Remarkably, W-boxes are also found in the promoter of *WRKY* genes, suggesting a potentially strong transcriptional networking between WRKY proteins.

**FIGURE 3:**
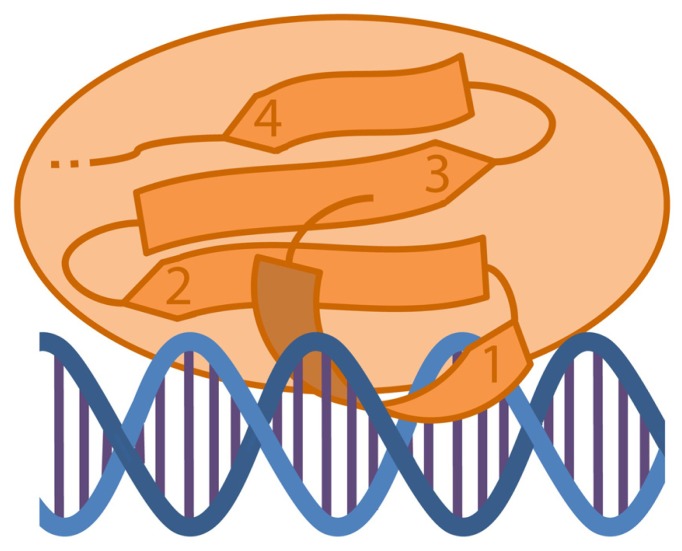
**Diagram of a WRKY C-terminal domain interacting with the DNA.** The C-terminal WRKY domain consists of a four-stranded antiparallel β-sheet (1–4). The DNA recognition takes place along the major grove by the β1-strand containing the WRKYQK motif.

The elucidation of the solution structure of WRKY proteins in contact with the DNA will help to understand the mechanism of DNA-binding. In 2005, the solution structure of the C-terminal WRKY domain of *Arabidopsis* WRKY4 (a group I WRKY protein) was discovered by NMR (Yamasaki et al., 2005). Yamasaki et al. (2012) could dissolve the structure of the same domain in complex with a W-box. The C-terminal WRKY domain consists of a four-stranded antiparallel β-sheet, in which the β1-strand, that comprises the WRKYGQK motif, contacts the major DNA groove (**Figure 3**). Residues of the WRKYGQK motif recognize the DNA mainly through apolar contacts with methyl groups of the T bases of the W-box. The DNA in this model is B-formed. Another model for the protein–DNA structure formation was proposed in 2007 by Duan et al. (2007). They investigated the crystal structure of the WRKY domain of Arabidopsis WRKY1 (also a group I WRKY protein), but the domain attribution used for WRKY1-C was longer. They found that this WRKY domain consists of a five-stranded antiparallel β-sheet with β2 and β3 forming the DNA-binding sites. The zinc-binding site was found between β4 and β5. By using a similar domain attribution like Yamasaki et al. (2012) the structure between WRKY4-C and WRKY1-C was comparable.

### THE W-box, SURROUNDING SEQUENCES AND THE WRKY DOMAIN DETERMINE THE DNA-BINDING SPECIFICITY

WRKY TFs bind W-boxes in the promoters of target genes to regulate their expression. But almost all WRKY factors bind W-boxes raising the question, how specificity is achieved between certain promoters and different WRKY TFs.

Binding studies revealed that only the presence of W-boxes is not sufficient for a DNA–protein interaction. By using gel shift experiments, Miao et al. (2004) could show a specific DNA-binding activity of WRKY53 to promoter fragments of its target gene *senescence-induced receptor-like kinase* in which a complex was only formed with a fragment containing four W-boxes, whereas no DNA–protein interaction was found when the fragment consisting of only three W-boxes. However, reporter gene assays showed, that these three boxes are necessary for the induction of a reporter gene by WRKY6 (Robatzek and Somssich, 2002). This indicates that the presence of W-boxes is not sufficient for specific binding and that most likely the surrounding sequences and the overall structures are important. A more detailed study for five different WRKY TFs toward their DNA-binding selectivity depending on neighboring sequences was performed by Ciolkowski et al. (2008). They found differences in the binding site preferences of WRKY6, WRKY11, WRKY26, WRKY38, and WRKY43 by gel shift experiments. WRKY6 (group IIb) and WRKY11 (group IId) show high binding affinity to sequences with a G residue directly adjacent 5′ to the W-boxes, whereas WRKY26 (group I), WRKY38 (group III), and WRKY43 (group IIc) bind more efficiently with a C, A, or T in the direct 5′ neighborhood. Interestingly, the binding of these three WRKYs was enhanced by exchanging the first T base in 5′ direction. Ciolkowski et al. (2008) concluded again that for a specific transcriptional regulation the adjacent sequences to W-boxes are important. Besides, there are some reports of WRKY proteins binding to non-W-box sequences. In a reporter gene assay, WRKY6 can regulate the reporter gene expression under the control of the *WRKY42* promoter lacking perfect W-boxes (Robatzek and Somssich, 2002). WRKY53 can also directly interact with a W-box lacking fragment of the same promoter (Miao et al., 2004) and with clustered imperfect W-boxes only consisting of the TGAC core elements of a W-box (Potschin et al., 2013) indicating more diversity in sequence affinities of WRKY TFs. Moreover, binding to a PRE4 element (TACTGCGCTTAGT) and to a W-box containing element was shown for *Os*WRKY13 of rice (Cai et al., 2008).

A DNA–protein interaction enzyme-linked immunosorbent assay (DPI-ELISA) screen was developed by Brand et al. (2013) to elucidate WRKY DNA-binding specificities in a more general view. They used only the WRKY DBDs for the DPI-ELISAs with the aim to unravel the DNA-sequence specificity for each WRKY DBD. The DBDs of *At*WRKY50, *At*WRKY11, and *At*WRKY33 (C-terminal DBD and N-terminal DBD) were tested and, in fact, they found sequences that seem to be DBD-specific. Remarkably, they could show that both DBDs of group I WRKYs are functional and can bind to DNA, even though the binding of the N-terminal DBD was weaker than that of the C-terminal DBD. Although homology modeling revealed a potential binding ability for both domains, the N-terminal domain always showed weaker or even no binding (Eulgem et al., 1999; Maeo et al., 2001; Duan et al., 2007). However, the actual function of the N-terminal WRKY domain is still unclear.

As mentioned above, there are sequence variants for the highly conserved WRKYGQK motif of the WRKY domain. The *Arabidopsis* WRKY50 factor has the slightly different amino acid sequence WRKYGKK in the WRKY domain (Eulgem et al., 2000; Brand et al., 2013). Brand et al. (2013) chose the WRKY domain of this WRKY TF and the *Arabidopsis* WRKY11 DBD with a conserved WRKYGQK motif to investigate, if there is a difference in the DNA recognition caused by this single amino acid exchange (lysine and glutamine) in the DNA-binding site. The amino acid glutamine prefers to bind nucleobases due to its partial negative charge, whereas lysine prefers to bind the phosphate backbone due to its partial positive charge. In fact, these WRKY domains showed preferences for distinct DNA target sequences, depending on this amino acid exchange in the conserved WRKYGQK motif. They mutated the conserved motif of WRKY50 to WRKYGQK^(KQ)^ and this of WRKY11 to WRKYGKK^(QK)^ and tested these mutated WRKY domain proteins in DPI-ELISAs. WRKY50^mut^ showed a similar DNA-binding affinity like WRKY11^wt^ and WRKY11^mut^ like WRKY50^wt^, suggesting that these amino acids in the WRKY domain are important for specific DNA recognition.

### W-boxes IN WRKY GENE PROMOTERS ENABLE TRANSCRIPTIONAL NETWORKING

An interesting point that has emerged in promoter analysis of WRKY TFs is the enrichment of W-boxes in their own promoters as indicated by Dong et al. (2003). They analyzed the 1.5 kb promoter sequence upstream of 72 WRKY genes in *Arabidopsis*, finding that 83% of the WRKY genes contain at least two perfect W-boxes (TTGACC/T) and 58% contain even four or more TTGAC core elements suggesting a regulatory network between the WRKY factors.

**FIGURE 4:**
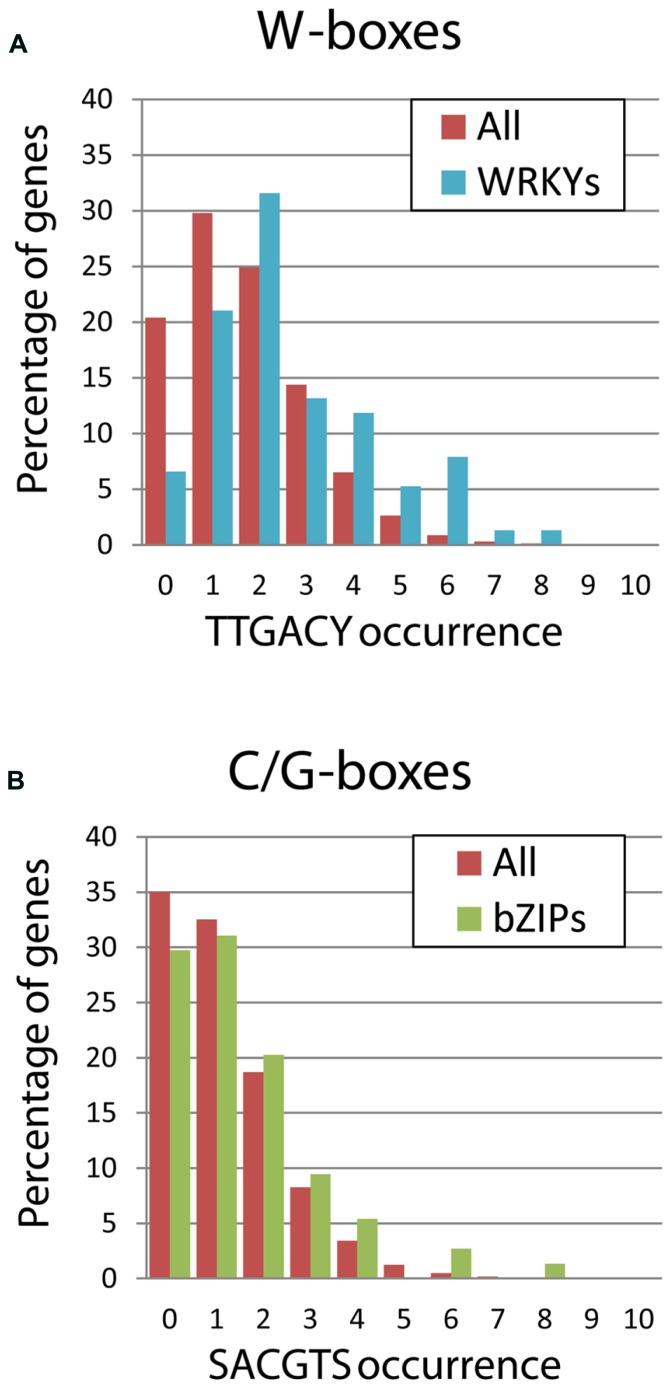
**Frequency of *cis*-element distribution within the 3-kb upstream of coding sequences.** The occurrence of the *cis*-elements was calculated only for the positive strand using the Patmatch tool in the TAIR website. Red bars refer to the whole set of 33,602 annotations of the TAIR10 Genome Release, while green and blue bars indicate the subsets including only bZIPs or only WRKYs genes, respectively. **(A)** The occurrence of W-boxes was determined using a TTGACY motif, where Y indicates pyrimidine. The enrichment of W-boxes in the WRKY promoters is remarkable, while only few WRKY promoters carry no W-boxes. **(B)** The occurrence of the G- and C-boxes, the preferred *cis*-elements bound by plant bZIPs, was determined using a SACGTS motif, where S indicate strong bases (C and G). In contrast, no increase of bZIP-binding sites in their promoters can be observed.

Further detailed studies of several WRKY promoters also confirmed the presence of multiple W-boxes. For example, two W-boxes were found in the promoter of *AtWRKY6*, four and five W-boxes in the promoters of the two homologous genes of *Coffea arabica*, five W-boxes in the promoter of *AtWRKY18*, and three perfect W-boxes plus an additional TGAC cluster in the promoter of *AtWRKY53* (Robatzek and Somssich, 2001; Petitot et al., 2013; Potschin et al., 2013). Some WRKYs even carry 11 or 12 (*AtWRKY66*, *AtWRKY17*) TTGAC core elements in an analyzed 1.5 kb promoter fragment (Dong et al., 2003). In order to compare the bZIP family with the WRKY family in this aspect, we analyzed bZIP promoters for C and G-boxes and WRKY promoters for W-boxes. We could easily verify this enrichment analyzing the 3-kb upstream fragments of 76 WRKY genes, which led to similar results: 72% of the WRKY genes contain two or more W-boxes. Furthermore, we found that 40% of the WRKYs have three or more W-boxes in their promoters, which is clearly above the average found for all annotations in the TAIR database (**Figure 4**). In contrast, no enrichment of C- or G-boxes could be detected in the bZIP promoters compared to the overall distribution of these *cis*-elements.

In agreement with the W-box enrichment in their own promoters, it has been demonstrated that the WRKY proteins act on the promoters of their own genes and on other WRKY genes in cotransfection assays resulting in activation or repression of a reporter gene (Robatzek and Somssich, 2002; Petitot et al., 2013; Potschin et al., 2013). In addition, a pull-down analysis of WRKY53 with genomic DNA resulted in a list of putative target genes of WRKY53 including eight different WRKY genes (Miao et al., 2004). Furthermore, the analysis of *wrky* mutant or overexpression plant lines revealed that the expression of other WRKY genes is altered in these lines. Loss of the *At*WRKY22 protein increased the expression of *AtWRKY70* after dark treatment, whereas overexpression of the *At*WRKY22 protein decreased the expression of *AtWRKY70* under normal conditions in comparison to wild type plants. When *AtWRKY70* is mutated, the expression of *AtWRKY22* is decreased compared to wild type plants after dark treatment (Zhou et al., 2011). Moreover, a double-knock out mutant of *Atwrky11 wrky17* showed increased transcript levels of *AtWRKY70* and *AtWRKY54* (Journot-Catalino et al., 2006). Microarray analyses of stressed *Atwrky33* mutant plants compared to the wild type revealed lower expression of *AtWRKY28*, which was also confirmed by qRT-PCR (Jiang and Deyholos, 2009). Alteration on the expression of certain WRKY genes was also shown for *Atwrky18* mutant compared to wild type plants in microarray analyses (Wang et al., 2006). Based on 2000 *Arabidopsis* microarray experiments, it was found that more than 70% (45 out of 61) of the WRKY genes are co-regulated with other WRKYs (Berri et al., 2009). In addition, ChIP resolution scanning of the parsley *PcWRKY1* promoter with an antiserum that detects most of the WRKY factors showed that the W-boxes of this promoter are constitutively occupied by WRKY factors (Turck et al., 2004). Therefore, it seems that the WRKY factors act in a network with mutually regulation of their own expression.

### WRKY PROTEINS CAN INTERACT WITH MULTIPLE PARTNERS

In addition to transcriptional networking, WRKY proteins can also form dimers and are also capable to form heterodimers. Furthermore, many other proteins have been characterized to form protein complexes with WRKY proteins thereby regulating their function. An excellent overview on protein interaction partners of WRKY proteins was recently published by Chi et al. (2013). Here, we focus on the heterodimer formation between WRKY factors and their impact on transcription.

The growing number of discovered interaction partners reveals that there is also a networking between the WRKY factors on the protein level. Moreover, there is some evidence that these WRKY heterodimers act in a different way on transcriptional regulation than homodimers or monomers. Recently, a participation of *At*WRKY18 in the senescence process was discovered (Potschin et al., 2013). *At*WRKY18 can physically interact with *At*WRKY53, an important regulator of early senescence, leading to different transcriptional activation in a reporter gene assay of the heterodimer in comparison to the single proteins. A well-investigated network exists between the three *Arabidopsis* WRKY factors WRKY18, WRKY40, and WRKY60. It was shown by Xu et al. (2006) that these three WRKYs interact with each other in a yeast two-hybrid assay and form homo- and heterodimers. In gel shift assays, WRKY18 and WRKY40 heterodimers bind much stronger to different W-box carrying sequences than the respective homodimers. In contrast, if WRKY40 is mixed with WRKY60 proteins the binding affinity declines. Since WRKY60 alone shows almost no binding activity for the used DNA sequences the effect has to be due to heterodimer formation. An example for the regulation activity of WRKY18/WRKY40 heterodimers is given by Chen et al. (2010). *WRKY60* is expressed after ABA treatment and this induction is almost lost in the *wrky18* and *wrky40* mutants, suggesting that *WRKY60* is regulated by WRKY18/WRKY40 in the ABA signaling pathway. In addition, they could show activation of the *WRKY60* promoter by WRKY18/WRKY40 heterodimers in a reporter gene assay, whereas the homodimers had no effect (Chen et al., 2010). These three WRKY proteins participate in the ABA signaling pathway through direct regulation of ABI4 and ABI5. Interestingly, not only different binding effects to these two genes were observed for the heterodimers, by using fragments of the ABI4 and ABI5 promoters in gel shift assays, binding activity of a combination of all three WRKYs together was sometimes completely abolished binding, although all possible heterodimers could bind to the same fragment. This indicates that an interaction between all three WRKY proteins takes place and that this higher order complex has again a distinct functionality (Liu et al., 2012).

An example of different binding activity for heterodimers between WRKYs and non-WRKY proteins is given by Lai et al. (2011). *At*WRKY33 can interact with SIGMA FACTOR-INTERACTING PROTEINS 1 and 2, two VQ motif-containing proteins that stimulate the DNA-binding activity of WRKY33. It was shown in a yeast two-hybrid assay using deletion mutants of WRKY33 that this interaction is mediated by the C-terminal WRKY domain of WRKY33. WRKY33 belongs to group I with the characteristic of two WRKY domains in which in general the C-terminal WRKY domain carries out the DNA-binding. However, the C-terminal WRKY domain is also responsible for mediating protein–protein interactions (Lai et al., 2011), so that these two functions overlap in this domain. Besides, in a yeast two-hybrid screen with *Arabidopsis* VQ and WRKY proteins, the C-terminal WRKY domain of group I WRKY proteins and the sole WRKY domain of group IIc WRKY proteins seem to be important for protein–protein interactions (Cheng et al., 2012). Group IIa WRKY proteins contain canonical LZ sequences and many other group II and III WRKYs have at least multiple leucine, isoleucine or valine residues at their N-termini, forming a similar structure of a LZ for protein–protein interactions (Chi et al., 2013).

W-boxes in the promoters of target genes are often clustered. Since one WRKY DBD is thought to bind one W-box, such W-box clusters in the DNA can mediate a complex formation of higher order protein complexes between different WRKY proteins. Depending on the orientation and the number of nucleotides between the W-boxes, the WRKY DNA-binding protein complex is composed of WRKY proteins with specific conformations. However, higher order complex formation does not only refer to clustered W-boxes, but also to separated W-boxes through DNA loop formations (Chi et al., 2013), enhancing again the variety of WRKY TF activity. In contrast to the bZIP factors that need to dimerize for DNA-binding, the mode of DNA-binding seems to be more diverse for the WRKY factors. They appear to bind as monomers, dimers or even as trimers (Xu et al., 2006; Ciolkowski et al., 2008; Liu et al., 2012). But although single WRKY proteins were usually used in gel shift experiments, it is still possible that these WRKYs form homodimers. The isolation of the solution structure of a WRKY protein in complex with DNA revealed that monomer binding occurs, although they did not use the whole protein for structure analysis.

### WRKY ACTIVITY IS ALSO MODULATED BY PHOSPHORYLATION BUT THROUGH DIFFERENT KINASES

As already described for bZIPs, WRKY TFs activity can also be modulated by phosphorylation. In the case of WRKYs, phosphorylation can be mediated through the mitogen-activated protein kinase (MAPK) pathway (Asai et al., 2002). Normally, a MAP kinase kinase kinase (MEKK) phosphorylates and activates a MAP kinase kinase (MKK) that in turn phosphorylates a MAPK responsible for phosphorylation and regulation of different effector proteins. An entire MAPK signaling cascade was characterized for the response of plant cells to the bacterial component flagellin which is sensed by the flagellin receptor FLS2 (flagellin-sensitive 2), a leucine-rich-repeat (LRR) receptor kinase. The MEKK MEKK1 is activated by the FLS2 kinase, MEKK1 activates MKK4/MKK5, two MKKs that activate MPK3/MPK6, two MAPKs that activate the effector proteins WRKY22/WRKY29 resulting in an immune response. Activation of this MAPK cascade confers resistance to both bacterial and fungal pathogens (Asai et al., 2002). Phosphorylation-dependent activation in immune responses was also shown for *Nicotiana benthamiana* WRKY8. *Nb*WRKY8 increases its DNA-binding activity after incubation with salicylic acid-induced protein kinase (SIPK), a MAPK that is also able to phosphorylate *Nb*WRKY8. Additionally, phosphorylation of *Nb*WRKY8 resulted in an enhanced transactivation activity in a reporter gene assay (Ishihama et al., 2011). For its homolog *At*WRKY33, a regulation through phosphorylation was also shown. Transcriptomic analysis of *wrky33* and wild type plants upon *Botrytis cinerea* infection discovered a strong transcriptional reprograming mediated by *At*WRKY33 in plant pathogen responses (Birkenbihl et al., 2012). *At*WRKY33 is a substrate of MPK3/MPK6, two MAPKs important for the induction of camalexin biosynthesis (Ren et al., 2008), the major phytoalexin in *Arabidopsis*, and is therefore responsible for growth inhibition of certain pathogens (Glawischnig, 2007). Mutation of five potential phosphorylation sites in WRKY33 in the *wrky33* mutant background blocks the ability of WRKY33 to restore the induction of camalexin production (Mao et al., 2011). *At*WRKY53, a positive regulator of senescence is phosphorylated by MEKK1 although this kinase is upstream in the MAPK signal cascade and appears to take a short cut. The phosphorylation enhances DNA-binding activity of *At*WRKY53 *in vitro* and transcription of a reporter gene *in vivo* (Miao et al., 2007). Phosphorylation is often mediated by clustered Pro-directed Ser residues (SP-cluster) in the N-terminal region of several group I WRKY proteins. In addition, some group I WRKYs harbor a so called D-domain [(K/R)_1-2_-x_2-6_-(L/I)-x-(L/I)] important for the interaction with MAPKs (Ishihama et al., 2011). However, interaction with MAPKs is not restricted to group I WRKY proteins. Popescu et al. (2009) found in protein microarrays a lot of WRKYs from different groups as interaction partners of diverse MAPKs with most of the WRKYs carrying SP-cluster.

### WRKY EXPRESSION IS ALSO UNDER EPIGENETIC CONTROL

In eukaryotic cells, nuclear DNA wraps around histone proteins forming nucleosomes that are finally packaged into chromatin. Whereas euchromatin is the loosely packaged form accessible for the transcription machinery, heterochromatin is tightly packaged and transcriptionally inactive. These two states are not static but can be converted into each other providing an essential mechanism of regulating gene expression. Conversions are predominantly achieved through modifications of the histones by acetylation, methylation, and phosphorylation. Acetylation of a histone results in a more loosely form of the nucleosome and an easier access of the transcription machinery for gene expression. This kind of modification is mediated by histone acetyltransferases, which add acetyl groups to activate gene expression, and histone deacetylases, which remove acetyl groups to inactivate gene expression. Kim et al. (2008) could show that *At*WRKY38 and *At*WRKY62, two negative regulators of plant defense, interact in the nucleus with histone deacetylase 19 (HDA19), a positive regulator of plant defense. Both WRKYs show transactivation activity in a reporter gene assay which is abolished by HDA19 suggesting that *At*WRKY38 and *At*WRKY62 induce the expression of genes negatively regulating plant defense and this is inhibited by HDA19. Epigenetic control was also observed for *AtWRKY53* during senescence. For this WRKY gene, specific histone methylations are necessary for correct gene expression and progression of senescence (Ay et al., 2009). Methylation of histones can either activate or repress transcription depending on the methylated site mediated by histone methyltransferases and histone demethylases. Plants overexpressing *SUVH2*, a histone methyltransferase, have a different status of histone methylation, whereby the expression of *AtWRKY53* is repressed (Ay et al., 2009). But also histone acetylation seems to be important for *AtWRKY53* expression since the promoters of *AtWRKY53* and *AtWRKY6* are enriched with acetyl groups (Luna et al., 2012). Recently, yeast two-hybrid and bimolecular fluorescence complementation assays showed that banana *Ma*WRKY1 could interact with *Ma*HIS1, a linker histone H1 protein (Wang et al., 2012).

### WRKY FUNCTION CAN BE TRIGGERED BY SUBCELLULAR LOCALIZATION

Most WRKY TFs are located in the nucleus for direct transcriptional regulation (Robatzek and Somssich, 2001; Zhang et al., 2004; Zheng et al., 2006; Liu et al., 2007b). However, an interesting example for WRKY TFs that regulate gene expression by changing their subcellular localization is given by Shang et al. (2010). Usually, WRKY40 inhibits expression of ABA-responsive genes in the nucleus. Triggered by high concentrations of ABA, *At*WRKY40 interacts strongly with magnesium-protoporphyrin IX chelatase H subunit [CHLH]/putative ABA receptor (ABAR) inhibiting further regulatory function of *At*WRKY40 in the nucleus. ABAR is localized predominantly in the outer chloroplast membrane, with its N- and C-terminus exposed to the cytosol. ABAR binds ABA and appears to be an ABA receptor (Shen et al., 2006). *At*WRKY40 interaction with the C-terminus of ABAR in the cytosol releases inhibition of ABA response genes in the nucleus and ABA response can occur. Furthermore, the expression of *AtWRKY40* is repressed after ABA treatment (Shang et al., 2010).

## CONCLUSION

**FIGURE 5:**
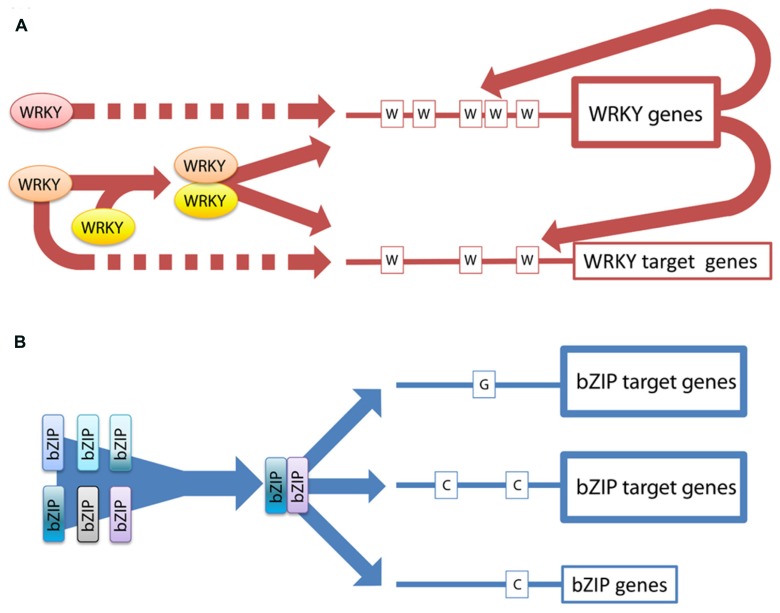
**Model featuring the main differences between the regulatory strategies of the two families. (A)** The expression of WRKY TFs is strongly regulated at the transcriptional level. The promoters of the WRKY genes are enriched in W-boxes, which are bound by WRKY proteins indicating transcriptional networking and also feed-back regulations. The dotted lines indicate that WRKY possibly interact with the DNA also in a monomeric stage. In addition, WRKYs can form dimers and thereby increase the variability in regulating specific target genes. **(B)** In the bZIP family heterodimerization is extensively used to increase variability in target gene regulation. The activity of the bZIPs is regulated via specific dimerization which determines the target specificity. There are no indications for transcriptional networking.

The hitherto characterized regulatory mechanisms controlling the function of TFs belonging to the bZIP and the WRKY families have been summarized in order to offer a comparative view. Not surprisingly, the major known mechanisms controlling protein activity have been found regulating members of both families. However, the prevalence of certain regulatory mechanisms reveals preferences in the manner how the activity of the proteins in each family is controlled, what we designate as a general regulatory strategy (**Figure 5**). In the case of the bZIPs, networking on the protein level by heterodimerization appears to be the preferred tool to adjust and fine-tune bZIP function. Regarding the WRKYs, controlling transcription of each other stands out as networking strategy for this family in synergism with the epigenetic control of their promoters.

It is tempting to speculate about the implications of using these different strategies. It can be argued that the bZIP strategy of heterodimerization with a strong component of post-translational regulation would enable very fast crosstalk between different input signals but at the same time imply keeping a pool of “ready to use” monomers. Such an energetically expensive strategy must grant counteracting advantages in order to be maintained during the evolution. Besides the fact that the bZIP strategy allows for rapid responses, factor combination confers enhanced integration capacity and flexibility: a limited pool of monomers allows multitudes of responses. The looseness of the dimerization guarantees a certain degree of graduation and fine-tuning of several responses at the same time. In another sense, the WRKY strategy seems to actively strive for autocontrol by a decisive regulation of the own expression. Although this strategy results in slower responses, for it requires *de novo* synthesis of proteins, it ensures the proper timing and the steadiness of the response. These kinds of responses are expected to be rather long-term ones, so that they become buffered once they have been triggered. In agreement with these conjectures, bZIPs seem to have a more prominent role regarding stress adaptation, which require dynamic, adaptive responses; whereas WRKYs are frequently related to longer lasting situations, like pathogen defense or the senescence progression.

To sum up, we suggest that bZIPs and WRKYs follow different regulatory strategies and we hypothesize that these reveal different control methods, either the “adjustable kind” or the “slow-but-sure” one. Although there are some facts which are undisputed, as the enrichment of WRKY binding sites in their own promoters, further data will be required to support our hypothesis. To this end, the identification and characterization of further response pathways involving WRKYs and bZIPs as well as system biology approaches combined with bioinformatics and modeling will help to unravel the network strategies of the two families in more depth. However, new *in vivo* approaches will be necessary to follow also the dynamic of these processes. In addition, deciphering molecular evolution of the two TF families in more detail might also provide inside into the strategies that these gene families pursue.

## Conflict of Interest Statement

The authors declare that the research was conducted in the absence of any commercial or financial relationships that could be construed as a potential conflict of interest.

## ABBREVIATIONS

ABAR: magnesium-protoporphyrin IX chelatase H subunit (CHLH)/putative ABA receptor
ABF: ABA responsive element binding factor
ABI: ABA-insensitive
AREB: abscisic acid responsive elements-binding protein
BBX25: B-box domain protein 25
bZIP: basic region/leucine zipper
CCA1: circadian clock associated 1
CKII: casein kinase II
COP1: constitutive photomorphogenic 1
EEL: enhanced em level
EmBP-2: ABRE-binding factor Embp-2 (*Zea mays*)
FLS2: flagellin-sensitive 2
GBF: G-box binding factor
HDA19: histone deacetylase 19
HIS1: histone H1
HY5: elongated hypocotyl 5y
HYH: HY5 homolog
ICS1: isochorismate synthase 1
LSD1: lesions simulating disease resistance 1
MEKK: MAP kinase kinase kinase
MKK: MAP kinase kinase
MPK: MAP kinase
NPR1: non-expressor of PR genes 1
OREB1: ABRE binding factor OREB1 (*Oryza sativa*)
PR1: pathogenesis related 1
RBCS1a: ribulose bisphosphate carboxylase small chain 1a
SIB: sigma factor-interacting protein
SIPK: salicylic acid-induced protein kinase
SnRK: SNF1-related protein kinase
*SUVH2*: su(var)3-9 homolog 2
TGA: TGA factor family
TRAB1: ABRE binding factor TRAB1 (*Oryza sativa*)
WRKY: WRKY transcription factor
YAP: yeast AP-1-like transcription factor
ZmBZ-1: bZIP transcription factor 1 (*Zea mays*)
